# Theoretical Prediction of the Anti-Icing Activity of Two-Dimensional Ice I

**DOI:** 10.3390/molecules28166145

**Published:** 2023-08-20

**Authors:** Sicheng Liu, Xiaoyan Liu, Yining Li, Qing Guo, Xiangting Yu, Yi Yin, Haoze Jing, Peng Zhang

**Affiliations:** School of Space Science and Physics, Shandong University, Weihai 264209, China; liusicheng@mail.sdu.edu.cn (S.L.); liuxiaoyan@mail.sdu.edu.cn (X.L.); yiningli@mail.sdu.edu.cn (Y.L.); qingguo@mail.sdu.edu.cn (Q.G.); yu_xiangting@163.com (X.Y.); yinyi@mail.sdu.edu.cn (Y.Y.); jhz0509@mail.sdu.edu.cn (H.J.)

**Keywords:** two-dimensional ice, anti icing, hydrophobic

## Abstract

Two-dimensional (2D) ice I is atomic-level ice that is composed of two interlocked atomic layers saturated with hydrogen bonds. It has recently been experimentally observed, but its properties have yet to be clarified. Accordingly, we theoretically studied the hydrophobic properties of 2D ice I. On the contrary, a simulation of a hydrogen fluoride molecule on a 2D ice surface manifested that it destroyed the 2D ice structure and connected new hydrogen bonds with water molecules. Investigations of the interfacial effect between 2D and three-dimensional (3D) ice films indicated that the network structure of 2D ice was not destroyed by a 3D ice surface, as the former was saturated with hydrogen bonds. However, the surface of 3D ice reorganized to form as many hydrogen bonds as possible. Thus, the 2D ice film was hydrophobic and inhibited the growth of 3D ice. This shows that if 2D ice can be produced on an industrial scale, it can be used as an anti-3D-icing agent under low temperatures.

## 1. Introduction

Water ice is a ubiquitous substance. More than 19 three-dimensional (3D) ice phases have been observed under extreme temperature and pressure conditions at a laboratory scale [1,2]. In addition, two-dimensional (2D) ice has recently attracted considerable research interest. In 1997, Koga proposed a double-layer ice structure based on molecular dynamics simulations [3]. In 2015, Molinero et al. reported the discovery of a tetragonal 2D ice phase in bilayer graphene [4]. In 2020, Jiang and co-workers described their use of high-resolution qPlus atomic microscopy to observe an interlocked 2D ice structure based on Au (111) that they named 2D ice I [5,6]. They found that 2D ice I is stably saturated with hydrogen bonds. So far, many methods have been used to detect the properties of ice, such as the X-ray method. These methods will have important applications in the detection of 2D ice properties [7,8,9,10]. Most of the research on the properties of 2D ice has been focused on its structural characteristics and electronic, dielectric, and optical properties [11,12]. This revealed that 2D ice phase I has an indirect band gap lattice and thus exhibits anisotropic optical properties. 

As an atomic-level material, 2D ice is likely to be of significance in the fields of material science, atmospheric science, and biology, among others [13,14,15,16,17,18]. However, the laboratory characterization of 2D ice is highly challenging, owing to the complexity of the experimental conditions involved. As such, we used the density functional theory (DFT), a quantum mechanical method, to simulate the interaction between a water monomer and 2D ice, and thus determined the adsorption properties of 2D ice.

The DFT was introduced by Professor Walter Kohn, which uses electron density instead of wave function to describe multi-electron systems [19,20]. Many similar works on the study of the adsorption and interface effects of 2D material surfaces have been reported. For example, Son et al. studied graphene oxide (GO) by constructing a model of organic molecules on its surface. They found that the oxidation reaction is likely to be carried out by transferring hydrogen atoms from the organic starting material to the surface of GO. They calculated the change in the system energy and showed that GO has a high chemical potential and a tendency to participate in chemical reactions during the process [21]. Fan et al. studied the adsorption of Li^+^ ions on the graphene surface. The Li^+^ ions were placed on the surface with different distances. It was found that the system was stable at the position of 1.84 Å [22]. Nakada systematically calculated the energy of the chemical element from Z = 1 to 84 at three adsorption sites on 3 × 3 graphene using the PAW method of DFT calculation [23]. Boukhvalov used the DFT method to model different types of graphene. Their static (substrate, shape, curvature, strain, and doping) and dynamic (starting point of functionalization, migration barriers, and stability of configurations) aspects were investigated, which provided model parameters for the adsorption on graphene substrates [24].

In this work, using DFT methods, we simulated the adsorption properties and interface effects between 2D ice and water monomer and 3D ice. The results indicate that 2D ice is hydrophobic and inhibits the growth of 3D ice. Thus, if 2D ice can be produced on an industrial scale, it would be a useful anti-icing agent and a valuable lubricant.

## 2. Results and Discussion

**Figure 1:**
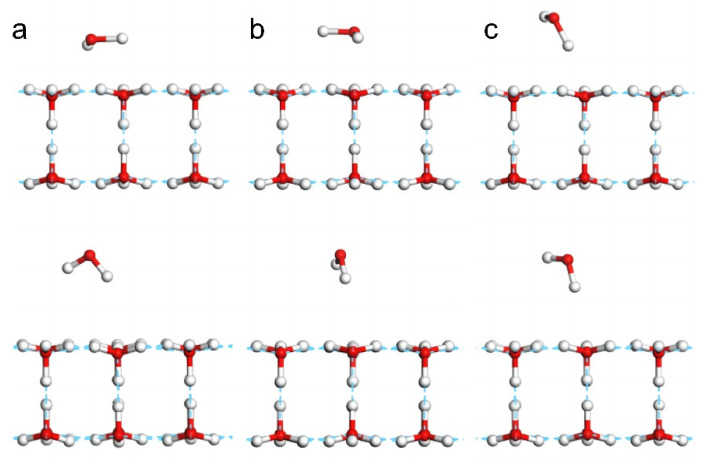
**Side view of three models involving a water molecule on the 2D ice surface.** (**a**,**b**) are horizontal configuration models, (**c**) is upright configuration model. The top row shows the constructed conformations, and the bottom row shows the corresponding optimized geometries. Red and grey balls represent oxygen and hydrogen atoms, respectively.

To demonstrate the hydrophobicity of 2D ice, we simulated the adsorption behavior of a water molecule on the 2D ice (001) surface to determine whether this monomer could break the 2D ice structure and construct new hydrogen bonds with the 2D ice film. As the length of a hydrogen bond is approximately 1.8 Å, the monomer was placed at approximately 1.9–2.5 Å above the 2D ice surface. We constructed three kinds of configurations by adjusting the monomer orientations, namely two horizontal configurations (Figure 1a,b) and one upright configuration (Figure 1c). Figure 1a shows the horizontal configuration in which a hydrogen atom of the monomer was placed on top of an oxygen atom on the ice surface. Figure 1b shows the horizontal configuration in which an O-H bond of the monomer was placed on top of an H-O bond on the ice surface. Figure 1c shows the upright configuration. After geometry optimization (bottom row of Figure 1), in each configuration, the monomer was repelled by the 2D ice and thus moved farther from the surface. Supplementary Material Video S1 demonstrates how the monomer disturbed the surface molecules and was ultimately repelled. The distances between the nearest hydrogen atom of the monomer and an oxygen atom on the ice surface changed from 1.995 Å to 2.728 Å (Figure 1a); from 1.995 Å to 3.299 Å (Figure 1b); and from 1.914 Å to 2.355 Å (Figure 1c), respectively. Clearly, the oxygen atom of the monomer exhibited more repulsive behavior than its two hydrogen atoms. 

After optimization, the total energies of the three systems depicted in Figure 1a–c were −30,545.874 eV, −30,545.872 eV, and −30,545.882 eV, respectively. The upright configuration (Figure 1c) exhibited the lowest potential, and the system energy was −30,544.857 eV before optimization. Thus, the energy of the system was reduced by 1 eV. This shows that the monomer could not break the 2D ice structure to form new hydrogen bonds. Supplementary Material Video S1 shows the process of water molecules being repelled by 2D ice.

**Figure 2:**
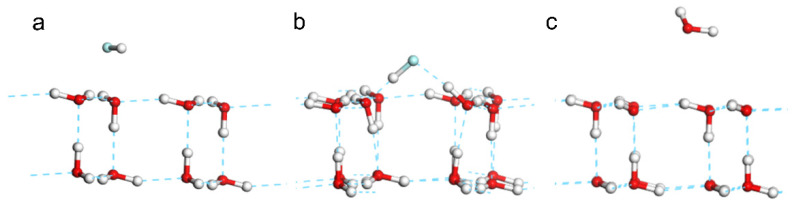
**Comparisons of adsorption effect of HF and H2O on 2D ice surface.** (**a**) The initial position of a HF molecule on the 2D ice surface and (**b**) the optimized result of the HF model. (**c**) The optimized result of an H2O molecule placed in the same position.

Two comparative models of HF and H2O monomers in parallel with one O-H bond of H2O at the 2D ice surface were optimized. The distance of the fluorine and hydrogen atoms was set as 1.927 Å. After geometry optimization, the HF molecule clearly broke the film structure and reformed two hydrogen bonds with two water molecules at the 2D ice surface (Figure 2b). On the contrary, the H2O monomer in the same position was repelled from 1.927 Å to 2.904 Å (Figure 2c). This is because the electronegativity of F is stronger than that of O, so F could break the hydrogen bond of 2D ice and reformed the hydrogen bond with it. This illustrates that HF is a good solvent to melt 2D ice. Supplementary Material Video S2 shows the process of 2D ice film being destroyed by a HF molecule.

**Figure 3:**
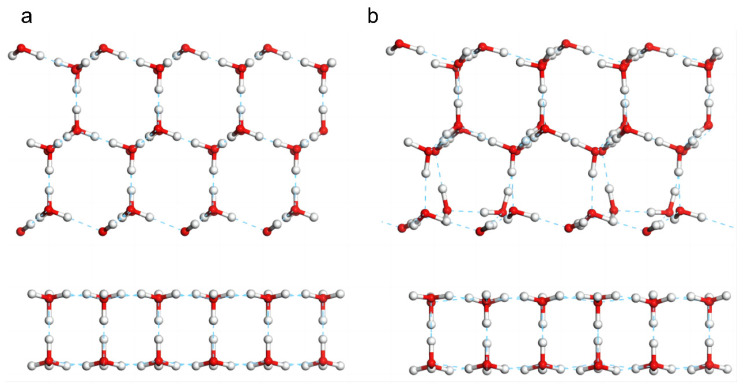
**Interfacial effect between ice Ic (111) surface and 2D ice (001) surface.** (**a**) Constructed model of 2D and 3D ice films and (**b**) geometry optimization results. The surface of 3D ice reorganized to form more hydrogen bonds while the 2D ice remained stable.

Subsequently, we studied the interfacial effect between 3D and 2D ice. To integrate the two types of films into one periodic cell, hydrogen-ordered ice Ic was used as the 3D ice because its lattice constant is well matched with that of 2D ice. The 3D ice film was superimposed onto the 2D ice (Figure 3a), and the geometry of the resulting system was optimized. Simulations were performed to explore whether 3D ice could destroy the stable hydrogen bond structure of the 2D ice and recombine to form a new 3D ice film. The result is shown in Figure 3b. There were no hydrogen bonds connecting the two layers. Because 2D ice was saturated with hydrogen bonds, the atomic-level film was highly stable. In contrast, because the 3D ice surface was not saturated with hydrogen bonds, the surface atoms encountered repulsive forces from the 2D ice and reorganized to form as many hydrogen bonds as possible. That is, 2D ice inhibited the growth of 3D ice on its surface. Supplementary Material Video S3 shows the dynamic process of this interfacial effect.

**Figure 4:**
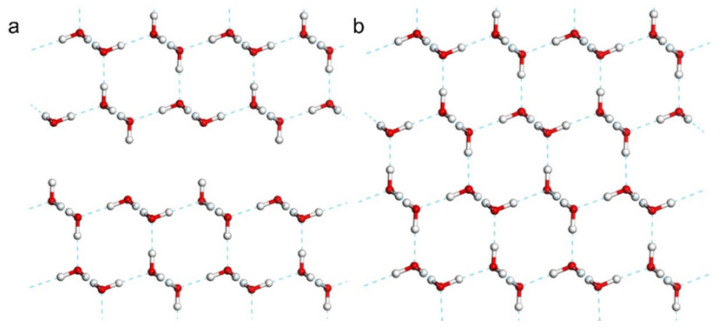
**Interfacial effect between two 3D ice films.** (**a**) Constructed model of two 3D ice films and (**b**) geometry optimization results.

The results of the simulation of the interfacial effect between two 3D ice films are shown in Figure 4. Although the two 3D ice films were placed in parallel at a large distance, they easily integrated through the formation of hydrogen bonds. This result was expected because 3D ice is hydrophilic and thus did not inhibit the growth of a new ice film on its surface (Supplementary Material Video S4 shows the effect). Comparing these two cases reveals that the 2D ice was hydrophobic. Because the 2D ice was saturated with hydrogen bonds, the interlocking hexagonal double-layer 2D ice film could not be destroyed by water molecules in the ambient environment. This demonstrates that 2D ice is a potential anti-icing agent. Furthermore, a multilayer framework composed of 2D ice is expected to be a promising lubricant, similar to graphite [25], for use in cold environments.

## 3. Simulation Strategy

The DFT code CASTEP was used to identify the geometries of water adsorption on 2D ice. The CASTEP program was originally developed in the Theory of Condensed Matter Group at Cambridge University, UK. It is a quantum mechanical code for electronic structure calculation based on the DFT method. Its advantage is that it can determine the ground state electronic structure of a system by solving the Schrödinger equation without using any experimental (empirical) data and only five basic constants (particle mass, charge, Planck constant, light speed, and Boltzmann constant), such as the band structure, optical properties, and mechanical properties. The basic method of using the CASTEP code to calculate the electronic structure is as follows: A set of Kohn–Sham equations of a single electron is calculated by using the plane wave approximation method. The energy and wave function of the single electron orbit are obtained, and the ground state energy of the electron system is calculated. Using the periodic boundary conditions and the Bloch theorem, the wave function is expanded into the plane wave basis set. The real potential energy inside the core is modeled by norm-conserving and Ultrasoft pseudopotential. The CASTEP code can be used to simulate a variety of materials, including crystalline solids, surfaces, molecules, liquids, and amorphous materials [26]. 

For the exchange correlation energy between electrons, DFT uses electron density instead of wave function as the basic variable, but this does not seem to solve the problem of calculating the complexity of the exchange correlation between electrons [27]. When Kohn and Sham introduced the KS equation, they also introduced the local-density approximation (LDA) functional. However, this method is more applicable for the system with a uniform electron density. As for the system with uneven electron distribution, the Generalized Gradient Approximation (GGA) functional is introduced to correct the local change in the electron density. The Perdew–Burke–Ernzerhof functional is the analytic fit of the numerical GGA, and the PBE is an improvement of the Perdew–Wang 1991 (PW91) functional [28], including an accurate description of the linear response of the uniform electron gas and a smoother potential [29].

Given the large fluctuations in the electron densities, we used the GGA method for geometry optimization. According to our work on 3D ice phases, the revised PBE functional is the optimal exchange–correlation functional for phonon calculation [30,31,32,33,34,35]. The self-consistent field tolerance was set as $1\times 10^{-6}$ eV/atom. Using an ultrasoft pseudopotential, the energy cut-off was set to 340 eV, and the k-point mesh was 3×3×1.

**Figure 5:**
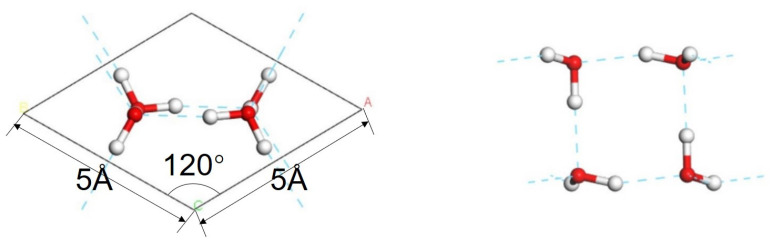
**Primitive cell of 2D ice I.** The left and right images show the top and side views, respectively.

With reference to the work of Jiang [5], we constructed a primitive cell of the 2D ice I structure, as shown in Figure 5. The lattice constants were a=b=5 Å,c=22.74 Å, α=β=90°,and γ=120°. To represent the 2D film, a large vacuum interval was set on the surface. Thus, the c-axis appeared to be rather long. In general, CASTEP simulations are based on periodic structures. To avoid the influence of the neighboring cells, we extended the 2D ice plane to a 4×4 supercell, i.e., a=b=20 Å. Then, we built a water monomer and copied it to the ice surface. Finally, three conformations were obtained, as shown in Figure 1. To compare the adsorption effect, a model of a hydrogen fluoride monomer placed on the 2D ice surface was constructed, too.

In the interfacial effect simulations, to match the coordinates of 2D ice, we selected ice Ic as the 3D ice sample. The lattice constants of ice Ic were set as follows: a=b=c=6.38 Å,c=22.7473 Å,and α=β=γ=90°. To match the lattice constants of the 2D ice, a film along the Ic (111) plane was cleaved with a thickness of four layers. Therefore, the parameters of the Ic film were adjusted as follows: a=b=15.6 Å and γ=120°. Subsequently, we expanded the 2D ice to a 3×3 supercell with the lattice constants of a=b=15 Å. Finally, we combined the 2D ice (001) film with the 3D ice Ic (111) film. In addition, we cleaved a bulk Ic sample into two samples placed approximately 3 Å apart and then performed geometry optimization as a comparative study.

## 4. Conclusions

Owing to the challenges in experimentally observing atomic-level 2D ice films [5], research on this material has been limited because it is difficult to prepare in laboratory environments. In this study, we used DFT to examine the hydrophobic properties of 2D ice. The dynamic analysis of the adsorption of a water monomer onto a 2D ice surface showed that the monomer could not break the 2D structure to form new hydrogen bonds. This hydrophobicity of the 2D ice was due to it being saturated with hydrogen bonds. On the contrary, the HF molecule may destroy the 2D ice film easily to show that HF is a good solvent to melt 2D ice.

An analysis of the interfacial effect between a 2D ice film and 3D ice showed that the surface of the 3D ice reorganized to form more hydrogen bonds when it was placed close to the 2D ice film. In contrast, two 3D ice films placed in parallel at a large distance from each other were easily connected by hydrogen bonds. These results suggest that 2D ice inhibits the growth of 3D ice. Therefore, 2D ice is a promising anti-icing material. In addition, a multilayer framework of 2D ice may exhibit excellent lubricating properties, similar to those of graphite. Experimental observations are expected to be made in the future.

## Data Availability

Not applicable.

## Supplementary Materials

The following supporting information can be downloaded at https://www.mdpi.com/article/10.3390/molecules28166145/s1, Video S1: Adsorption of H_2_O monomer on 2D ice surface. Video S2: Adsorption of HF monomer on 2D ice surface. Video S3: The interfacial effect between 3D ice and 2D ice. Video S4: The interfacial effect between two 3D ice films.

## Sample Availability

Samples of the compounds are available from the authors.

## References

[1] Millot M., Coppari F., Rygg J.R., Correa Barrios A., Hamel S., Swift D.C., Eggert J.H. (2019). Nanosecond X-ray diffraction of shock-compressed superionic water ice. Nature.

[2] Gasser T.M., Thoeny A.V., Fortes A.D., Loerting T. (2021). Structural characterization of ice XIX as the second polymorph related to ice VI. Nat. Commun..

[3] Koga K., Zeng X.C., Tanaka H. (1997). Freezing of confined water: A bilayer ice phase in hydrophobic nanopores. Phys. Rev. Lett..

[4] Moore E.B., Molinero V. (2011). Is it cubic? Ice crystallization from deeply supercooled water. Phys. Chem. Chem. Phys..

[5] Ma R., Cao D., Zhu C., Tian Y., Peng J., Guo J., Chen J., Li X.-Z., Francisco J.S., Zeng X.C. (2020). Atomic imaging of the edge structure and growth of a two-dimensional hexagonal ice. Nature.

[6] Guo J., Meng X., Chen J., Peng J., Sheng J., Li X.-Z., Xu L., Shi J.-R., Wang E., Jiang Y. (2014). Real-space imaging of interfacial water with submolecular resolution. Nat. Mater..

[7] Zhu Y., Wang F., Bai J., Zeng X.C., Wu H. (2015). Compression limit of two-dimensional water constrained in graphene nanocapillaries. ACS Nano.

[8] Bragg W. (1921). The crystal structure of ice. Proc. Phys. Soc. Lond..

[9] Whalley E. (1977). A detailed assignment of the O–H stretching bands of ice I. Can. J. Chem..

[10] Perakis F., Hamm P. (2012). Two-dimensional infrared spectroscopy of neat ice Ih. Phys. Chem. Chem. Phys..

[11] Ghasemi S., Alihosseini M., Peymanirad F., Jalali H., Ketabi S., Khoeini F., Neek-Amal M. (2020). Electronic, dielectric, and optical properties of two-dimensional and bulk ice: A multiscale simulation study. Phys. Rev. B.

[12] Chen J., Schusteritsch G., Pickard C.J., Salzmann C.G., Michaelides A. (2016). Two dimensional ice from first principles: Structures and phase transitions. Phys. Rev. Lett..

[13] Kiselev A., Bachmann F., Pedevilla P., Cox S.J., Michaelides A., Gerthsen D., Leisner T. (2017). Active sites in heterogeneous ice nucleation—The example of K-rich feldspars. Science.

[14] Zheng S., Li C., Fu Q., Hu W., Xiang T., Wang Q., Du M., Liu X., Chen Z. (2016). Development of stable superhydrophobic coatings on aluminum surface for corrosion-resistant, self-cleaning, and anti-icing applications. Mater. Des..

[15] Murray B.J., Knopf D.A., Bertram A.K. (2005). The formation of cubic ice under conditions relevant to Earth’s atmosphere. Nature.

[16] Atkinson J.D., Murray B.J., Woodhouse M.T., Whale T.F., Baustian K.J., Carslaw K.S., Dobbie S., O’Sullivan D., Malkin T.L. (2013). The importance of feldspar for ice nucleation by mineral dust in mixed-phase clouds. Nature.

[17] Liou Y.-C., Tocilj A., Davies P.L., Jia Z. (2000). Mimicry of ice structure by surface hydroxyls and water of a β-helix antifreeze protein. Nature.

[18] Hetzel R., Hampel A. (2005). Slip rate variations on normal faults during glacial–interglacial changes in surface loads. Nature.

[19] Hohenberg P., Kohn W. (1964). Inhomogeneous electron gas. Phys. Rev..

[20] Kohn W., Sham L.J. (1965). Self-consistent equations including exchange and correlation effects. Phys. Rev..

[21] Boukhvalov D.W., Dreyer D.R., Bielawski C.W., Son Y.W. (2012). A computational investigation of the catalytic properties of graphene oxide: Exploring mechanisms by using DFT methods. ChemCatChem.

[22] Zheng J., Ren Z., Guo P., Fang L., Fan J. (2011). Diffusion of Li+ ion on graphene: A DFT study. Appl. Surf. Sci..

[23] Nakada K., Ishii A. (2011). Migration of adatom adsorption on graphene using DFT calculation. Solid State Commun..

[24] Geim A.K. (2009). Graphene: Status and prospects. Science.

[25] Boukhvalov D. (2013). DFT modeling of the covalent functionalization of graphene: From ideal to realistic models. Rsc Adv..

[26] Clark S.J., Segall M.D., Pickard C.J., Hasnip P.J., Probert M.I., Refson K., Payne M.C. (2005). First principles methods using CASTEP. Z. Für Krist.-Cryst. Mater..

[27] Segall M., Lindan P.J., Probert M.A., Pickard C.J., Hasnip P.J., Clark S., Payne M. (2002). First-principles simulation: Ideas, illustrations and the CASTEP code. J. Phys. Condens. Matter.

[28] Perdew J.P., Ziesche P., Eschrig H. (1991). Electronic Structure of Solids’ 91.

[29] Perdew J.P., Burke K., Ernzerhof M. (1996). Generalized gradient approximation made simple. Phys. Rev. Lett..

[30] Yuan X.-Q., Yu X.-H., Zhu X.-L., Wang X.-C., Liu X.-Y., Cao J.-W., Qin X.-L., Zhang P. (2022). Comparative analysis of the hydrogen bond vibrations of ice XII. ACS Omega.

[31] Ning S.-Y., Cao J.-W., Liu X.-Y., Wu H.-J., Yuan X.-Q., Dong X.-T., Li Y.-N., Jiang Y., Zhang P. (2022). Computational Analysis of Hydrogen Bond Vibrations of Ice III in the Far-Infrared Band. Crystals.

[32] Liu X.-Y., Cao J.-W., Qin X.-L., Zhu X.-L., Yu X.-H., Wang X.-C., Yuan X.-Q., Liu Y.-H., Wang Y., Zhang P. (2021). A Computational Validation of water Molecules Adsorption on an NaCl surface. Crystals.

[33] Dong X.-T., Qin X.-L., Wang X.-C., Cao J.-W., Liu X.-Y., Yu X.-H., Yuan X.-Q., Guo Q., Sun Y., Zhang P. (2022). Computer simulation of hypothetical hydrogen ordered structure of ice XIX. Phys. Chem. Chem. Phys..

[34] Xiao-Ling Q., Xu-Liang Z., Jing-Wen C., Hao-Cheng W., Peng Z. (2021). Investigation of hydrogen bond vibrations of ice. Acta Phys. Sin..

[35] Zhu X.-L., Cao J.-W., Qin X.-L., Jiang L., Gu Y., Wang H.-C., Liu Y., Kolesnikov A.I., Zhang P. (2019). Origin of two distinct peaks of ice in the THz region and its application for natural gas hydrate dissociation. J. Phys. Chem. C.

